# Effect of Choice of Flavor of Fluoride Varnish on Behavior in Dental Visits in Schoolchildren

**DOI:** 10.1002/cre2.70069

**Published:** 2025-02-03

**Authors:** R. Karim, C. H. Splieth, J. Schmoeckel

**Affiliations:** ^1^ Department of Paediatric Dentistry University of Greifswald Greifswald Germany

**Keywords:** dental behavior, dental cooperation, fluoride flavors, fluoride varnish

## Abstract

**Objective:**

To investigate the effect of choosing the taste of a fluoride varnish on the behavior and acceptance of the children during the dental visit.

**Material and Methods:**

This single‐blinded, randomized controlled trial (NCT05285228) involved 70 healthy children aged 5–10 years who presented for a dental recall visit including the indication of an application of fluoride varnish to the specialized pediatric university dental service. The control group received the fluoride varnish (Profluorid varnish, VOCO Germany) with an allocated taste, whereas the test group had to choose the taste of the fluoride varnish just before the dental check‐up.

**Results:**

The vast majority of the children (*n* = 53, 75.7%) felt *happy/very happy* (Facial image scale) regarding the taste of the fluoride varnish, with no significant difference between both groups (*p* = 0.188). Dental behavior (Frankl behavior rating scale) was assessed generally as *positive/definitely positive* (*n* = 58, 82.2%). Interestingly, children with a history of previous negative dental behavior in the test group showed a tendency of more positive behavior than in the control group (66.6% vs. 33.4%, *p* = 0.244).

**Conclusion:**

Sense of control performed via choosing the flavor of the fluoride varnish increases the chance for positive behavior during the dental visit, especially in children with a history of negative dental behavior. Concurrently, it improves the child's taste acceptance, which is important for dental caries prevention.

**Trial Registration:**

The study protocol was registered on Cli ClinicalTrials.gov (NCT05285228).

## 1 Introduction

Dental anxiety is recognized as a major public health dilemma facing pediatric dentists in daily practice. Based on a previous German population survey an overall incidence of 11% of dental phobia has been reported (Enkling, Marwinski, and Jöhren 2006) which matches the overall global prevalence of early childhood dental fear and anxiety according to a recent systematic review (Sun et al. 2024). Children encounter dental anxiety independent of their age, sex and behavior management techniques (BMTs) is used. Hence, dental anxiety reduces or limits dental cooperation during dental visit, which then likely effects oral health‐related quality of life (Melebari, Attas, and Arafa 2019).

Sense of control is a common and well‐known noninvasive basic behavioral management technique for reducing disruptive behavior, which involves individuals' belief about their control in dealing certain events during certain treatments (Felicia, Satiadarma, and Subroto 2022). Still, in literature little evidence can be found for its effectiveness. To encourage children to share control, for example, by raising their hands and giving the opportunity to pause the dental procedures may increase the behavior in dental treatment. In addition, another successful approach is to give children the opportunity to choose from different materials such as the type or color of restorations placed on their teeth (Tyagi and Sharma 2011), and refers to the techniques of “locus of control” and at the same time providing “pseudo‐alternatives.”

A study evaluating the motivational effects of multicolor restorations on anxiety levels in pediatric patients and its motivational effects on oral health status found that the use improves the behavior in younger age groups (Melebari, Attas, and Arafa 2019). This emphasizes that the availability of choices of a specific material may create a more positive attitude towards treatment and reduce anxiety at the same time (Croll, Helpin, and Donly 2004).

Current evidence‐based recommendations for children with high caries risk include educating parents on daily oral hygiene, supervised tooth brushing with a 0.5% fluoride toothpaste/gel at home, dietary sugar‐intake control and attendance to the dental office for a regular recall every 3 months. This also involves an in‐office application of high‐dose fluoride varnishes and fissure sealants for erupting molars for caries prevention and, for example, silver diamine fluoride on cavitated lesions (American Academy of Pediatric Dentistry 2023).

It has been noted that taste preferences for sweet, salty, and umami are genetic, with an inherent rejection of bitter and sour tastes (Ventura and Mennella 2011). Taste preference in children is encouraged by children's innate liking for sweetness, in which bitterness is mainly associated with the presence of natural toxins, resulting low acceptance in children (Dovey et al. 2008). In addition to the evolutionary safety principle of not eating toxic foods, there is an innate aversion to unfamiliar tastes. Such neophobia is especially pronounced in infants (Macintyre 2014). Changes in taste preferences may occur prenatally or may be based on early childhood experiences. The effects of maternal diet during pregnancy and lactation on children's taste preferences were observed (Mennella et al. 2009; Beauchamp and Mennella 2011). Taste preferences after the first months of life are also influenced by sociocultural learning processes and social environment (Ellrott et al. 2007). The behavior of the parents and peers can influence this conditioning process in children. For example, receptivity to unfamiliar food tastes can be increased by peer social influence (Savage, Fisher, and Birch 2007).

The main assumption of the study is that when the child is given the option to choose the preferred taste of the fluoride varnish, there will be a positive effect on the acceptance and more importantly on behavior during the dental visit. Hence, the aim of this RCT is to investigate the impact of participation via choosing the taste of the fluoride varnish (Sense of control and pseudo‐alternative) on the taste acceptance and dental behavior during dental visits in children aged 5–10 years.

## 2 Materials and Methods

### 2.1 Study Design

This two‐parallel arm single‐blinded randomized controlled clinical trial was conducted at the Department of Paediatric Dentistry of the University Medicine Greifswald from February to July 2022 after ethical approval was obtained from the ethics committee of University Medicine Greifswald (Ethical approval number BB 180/21, 2022). The study was registered before recruitment on the website clinicaltrails.gov with the number (NCT05285228). The study was monitored and audited by the Department of Paediatric Dentistry of University Medicine Greifswald. Written informed consent was obtained from parents/caregivers of the child participants and also verbally from the participating child. After assessment of eligibility and acquiring informed consent, the child was randomly allocated using a hidden list with the randomized sequence.

At this stage (before the examination and the preventive session starts) fluoride varnish was placed by the nurse on the dental tray which was either chosen by the child or the randomly assigned flavor. This fluoride varnish was to be used at the end of the dental session. After allocation, the independent observer (KR) entered the dental treatment room for blinded assessment of the primary outcome variable “dental behavior” according to the Frankl behavior rating scale (FBRS) as shown in Table 1 (Frankl 1962). After the dental visit, the participating child, the accompanying parent or caregiver, the dental operator and the blinded assessor received a short questionnaire to be filled out.

**Table 1:** Frankl behavior rating scale for assessment of study's primary outcome (Frankl 1962).

Frankl behavior rating scales (FBRS)	Descriptions
**Definitely negative (1)**	Refusal of treatment, crying forcefully, fearful, or any other overt evidence of extreme negativism.
**Negative (2)**	Reluctant to accept treatment, uncooperative, some evidence of negative attitude but not pronounced, i.e., sullen, withdrawn.
**Positive (3)**	Acceptance of treatment, at times cautious, willingness to comply with the dentist, at times with reservation, but patient follows the dentist's directions cooperatively.
**Definitely positive (4)**	Good rapport with the dentist, interested in the dental procedures, laughing and enjoying the situation

The following inclusion and exclusion criteria were used in this study:

i. **Inclusion criteria**

- Children who are healthy or are Grade I or II according to ASAs (American Society of Anaesthesiologists) physical classification system, who present for a dental check‐up based on caries risk assessment in the previous visit (high caries risk: every 3 months vs. low caries risk: every 6 months) in which an application of fluoride varnish is indicated based on German oral preventive program within health insurance (Kassenzahnärztliche vereinigung 2023).
- Children aged between 5 and 10 years.
- Parental and child‐acquired consent for the child's participation in the study.

ii. **Exclusion criteria**

- Medically compromised patients who are Grade III, IV, or V according to ASA classification.
- Children who present to the appointment with pain and discomfort, which should be treated.
- Children who refuse to participate in the study.
- Children who present for the first time in the dental clinic, where no dental visit in the pediatric department have been done.
- Children who report an allergy against fluoride varnish.

**Figure 1:**
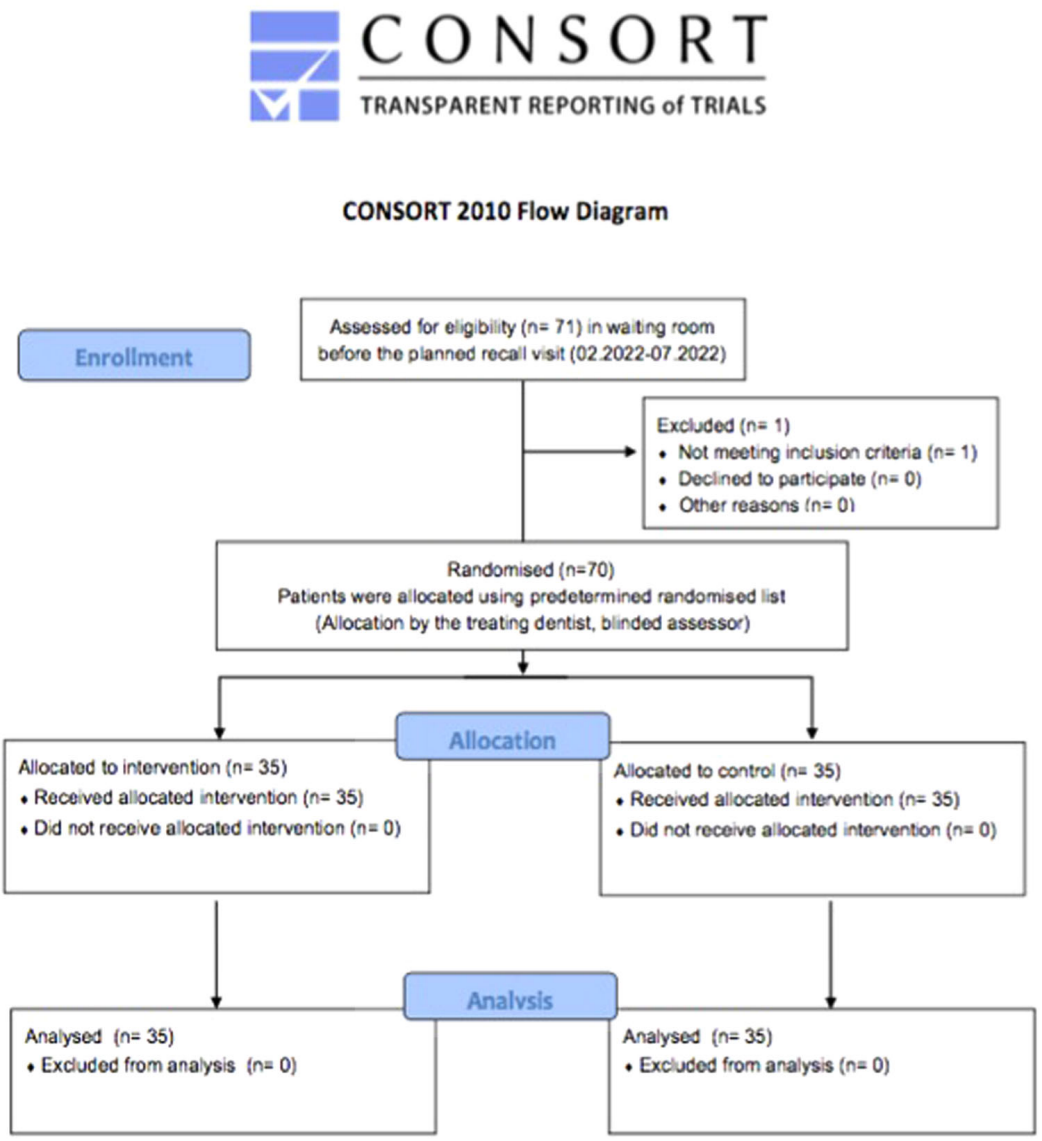
CONSORT flow diagram for this randomized, controlled trial depicting participants' enrolment in the study.

Seventy‐one children were reviewed for inclusion in this study from those being scheduled for check‐up/prophylaxis independently of the study and asked in the waiting room to voluntarily participate. None of the children disagreed to participate (participation rate 100%). The patient profile in the department consisted of many children with history of negative dental behavior based on their dental records (compare study sample: *n* = 27/70, 39%), and children with moderate to high caries risk based on dmft‐index (*n* = 35, 50%). Moreover, a large part of the recall patients had a history of invasive dental procedures (compare; *n* = 32, 45.7%; as shown in Table 2). One child was excluded afterwards for not meeting the inclusion criteria. The final convenience sample comprised 70 children, who were randomly assigned into two groups as shown in Figure 1.

**Table 2:** Baseline characteristics of the control and test groups (children; *n* = 70).

Category	Study groups (*n* = 70)		Total	Stat.
	Control *n* = 35 (50.0%)	Test *n* = 35 (50.0%)	*n* = 70 (100%)	*p*‐value
**Age (years)**				0.260^a^
Mean ( ± SD)	6.9 (± 1.6)	7.3 (± 1.7)	7.1 (± 1.6)	
Min.–Max.	5–10 (age = 5; *n* = 7)	5–10 (age = 5; *n* = 8)	5–10 (age = 5; *n* = 15)	
**Sex**				0.461^b^
Male	20 (57.1%)	23 (65.7%)	43 (61.4%)	
Female	15 (42.9%)	12 (34.3%)	27 (38.6%)	
**History of negative dental behavior** (dental records)	11 (31.4%)	16 (45.7%)	27 (39.0%)	0.220^b^
**History of invasive dental procedures** (dental records)	19 (21.4%)	22 (24.3%)	41 (24.3%)	0.631^b^
**Mean dmft** (dmft ± SD)	4.03 (± 3.50)	4.09 (± 3.50)	4.06 (± 3.49)	0.944^a^
Min.–Max.	0–14 (dmft < 4; *n* = 19)	0–9 (dmft < 4; *n* = 16)	0–14 (dmft < 4;*n* = 35)	
**Mean DMFT** (DMFT ± SD)	0.23 (± 0.73)	0.34 (± 0.73)	0.29 (± 0.73)	0.513^a^
Min.–Max.	0‐4 (DMFT > 2;*n* = 1)	0‐3 (DMFT > 2;*n* = 1)	0‐4 (DMFT > 2;*n* = 2)	

^a^ Two sample independent *t*‐test.

^b^ Chi‐square test.

### 2.2 Sample Size Calculation

The sample size was calculated with the program G*power version 3.1 on a two‐sample comparison of proportions of behaviors; one group with possibility of choosing the flavor of fluoride varnish (test group) vs allocated flavor of fluoride varnish (control group). The inclusion of 33 children in each group would be sufficient to detect a statistically significant difference with a power of 80% between interventions measured assuming a difference of 55% (control group) vs 85% (test group) in proportions. Considering the paired nature of the data for the primary outcome “behavioral assessment using FBRS” (Al‐Khotani, Bello, and Christidis 2016). With a slight overestimation of about 10% to allow for withdrawal of the study, the final sample in each arm was set at 35 children for each group (total: *n* = 70 children).

### 2.3 Randomization

Randomization was performed using a preformed allocations list (random.org). For blinding purpose, this list was only accessible to the dental nurse assisting in the dental session, in which the fluoride varnish was applied. This list was created with the use of random table method, in which 35 randomly generated serial numbers for each group were generated by Windows Microsoft Excel (2010, USA) to ensure equal randomized distribution to each group. Depending on the allocation, the following procedure was performed:

For the control group, the routine check‐up was performed with an application of fluoride varnish with 22,600 ppm fluoride (Profluorid VOCO, Germany) with a randomly predetermined taste using table randomization from five given tastes prior the session.

### 2.4 Materials

This study involved an application of fluoride varnish with 22,600 ppm fluoride (Profluorid Varnish, VOCO‐Germany) with either a randomly predetermined taste (control group) or self‐chosen taste (test group) during the dental session from a range of five flavors (melon, caramel, cherry, bubble gum, mint; Profluorid VOCO, Germany).

### 2.5 Intervention

Children were seated in a dental chair using standard infection control guidelines for a dental examination used at the department. The study and its objectives were explained to the children and their caregivers. In both study groups, the “Tell‐show‐do” conditioning technique according to the department's standard procedures (Appendix I) was used. The average time of the dental visit is 25‐30 min as children are scheduled every half an hour. The recall visit involved usual clinic examination, oral hygiene assessment using plaque disclosing agent (Mira 2‐Ton, Hager & Werken, Germany) and dental prophylaxis with the use of a slow‐speed rotary instrument with Robinson brush and highly concentrated fluoride gel (12,500 ppm, Elmex Gelee, CP GAPA, Germany).

To reduce the risk of bias, practitioners who performed the dental treatment were either pediatric specialists working at the Department of Paediatric Dentistry of the University Medicine Greifswald or postgraduate students undergoing a 3‐year master program in which there is an extensive theoretical and practical training. Hence, 3 specialist dentists and 4 postgraduate master students treated the children, who were recruited in this study.

### 2.6 Assessment Tools

- Primary Outcome: The overall participants' dental behavior during the study's dental visit was assessed using Frankl's Behavior Rating Scale (FBRS) by a single independent blinded observer (R.K.). This scale divides the behavior into four categories according to the Guideline on Behavior Guidance for the Paediatric Dental Patient on the basis of the behavior of children during the dental visit as shown in Table 1 (Frankl 1962). The proportions of negative (rating 1 and 2) vs. positive (rating 3 and 4) behavior were compared.
- The taste acceptance of the child was assessed using the facial image scale (FIS) consists of a row of five gray scale faces ranging from very happy to very unhappy, and each image has a score from 1 to 5 where 1 represents the most positive response and 5 represents the most negative response. Each participating child was asked to choose the face that represents his feeling at that instant right after application of the fluoride varnish (Alwin, Murray, and Britton 1991).
- A questionnaire was filled out by the caregivers (including questions on demographic issues) on their acceptance and preference regarding the choice of the taste of the fluoride varnish.
- Further data of the child for better description of the baseline characteristics of the participants was taken from the documentation in the patient's file (Dampsoft, Germany): for example, age, sex, dmft/DMFT, history of negative dental behavior, history of invasive dental treatment, for example, nitrous oxide sedation, extractions, and spacemainters (fixed and removable).
- History of negative dental behavior during previous dental appointments was reviewed from patient's dental records (Dampsoft, Germany) based on the following parameters in their records: sitting on parent's lap during the treatment, crying throughout the treatment and refusal of the entire treatment or a specific treatment step.

The study examiner (R.K.) was trained by two pediatric dentistry experts (J.S. and C.S.) to evaluate behavior according to the Frankl behavior rating scale (Frankl 1962). Training and calibration were performed through previously selected videos of 10 children showing different behaviors during recall appointments, which included oral examination and dental prophylactic in the dental environment. The assessments were performed at two different time periods, 10 days apart. The inter‐examiner kappa value was (Cohen's Kappa = 0.70), which is required for sufficient reliability level.

For the test group, before the standard check‐up starts, the participant was given the free choice of selecting the taste of the fluoride varnish from a range of five flavors (Melon, Caramel, Cherry, Bubble gum, Mint; Profluorid VOCO, Germany), which was applied at the end of the visit.

Finally, post‐placement instructions for both study groups provided by the manufacturer were given to the caregiver and the child and questionnaires were handed out.

### 2.7 Statistical Analysis

Child records were collected on a spreadsheet using Microsoft Excel 2013 and then analyzed first descriptively. Means and standard deviation were also calculated. Differences in child parameters (age, dmft, etc.) and clinical outcomes (dental behavior, acceptance, etc.) were analyzed depending on the type and distribution of data, using the chi‐square test, students *t*‐test, Fisher's exact test, and ANOVA. All analyses were performed in Microsoft Excel 2013. In this study, a value of *p* < 0.05 was defined to be statically significant.

## 3 Results

A total number of 70 children participated in this study analysis between February and July 2022. The mean age of the children was 7.11 ( ± 1.69) years. There was a higher proportion of male (*n* = 43) than female (*n* = 27) participants. The test group had a slightly higher number of children with a history of negative dental behavior according to previous dental records (*n* = 16: 45.7%) than the control group (*n* = 11; 31.4%). Whereas the control group had slightly lower mean DMFT compared to test group. Still, all differences in baseline characteristics between both groups were not statistically significant (Table 2).

Moreover, more than half of children in the study group (62.8%) chose either watermelon or cherry (each *n* = 11), followed by bubble gum (*n* = 7) and caramel (*n* = 5). However, mint taste was only chosen once (Table 3).

**Table 3:** Distribution of various flavors of fluoride varnish (Proflourid, VOCO; Germany) used in the study groups (*n* = 70).

Flavor of fluoride varnish	Study groups (*n* = 70)		Total
	Control *n* = 35 (50.0%)	Test *n* = 35 (50.0%)	*n* = 70 (100%)
**Watermelon**	7 (20.0%)	11 (31.4%)	18 (25.7%)
**Cherry**	7 (20.0%)	11 (31.4%)	18 (25.7%)
**Bubble gum**	7 (20.0%)	7 (20.0%)	14 (20.0%)
**Caramel**	7 (20.0%)	5 (14.3%)	12 (17.1%)
**Mint**	7 (20.0%)	1 (2.9%)	8 (11.4%)

FBRS assessment at the dental visit (by a blinded assessor) revealed positive behavior with statistically insignificant difference between the test and the control groups (*p* = 0.376). The majority of children (*n* = 58; 82.8%) in both study groups behaved positively during the dental visit. Though the control group (*n* = 7) had a slightly higher proportion of children behaving negatively than the test group (*n* = 5) as shown in Table 4.

**Table 4:** Behavior of the children during dental check‐up according to FBRS (blinded assessment, *n* = 70).

Frankl Behavior Rating Scale (FBRS)	Study groups		Total	Fisher exact test
	Control *n* = 35 (50%)	Test *n* = 35 (50%)	*n* = 70 (100%)	*p*‐value
**Definitely negative (1)**	0 (0.0%)	0 (0.0%)	0 (0.0%)	*p* = 0.376
**Negative (2)**	7 (20.0%)	5 (14.3%)	12 (17.1%)	
**Positive (3)**	10 (28.6%)	12 (34.3%)	22 (31.4%)	
**Definitely positive (4)**	18 (51.4%)	18 (51.4%)	36 (51.4%)	

Due to partially small numbers in cells, stat. sign. was assessed with the cut‐off between negative (1 and 2) vs. positive (3 and 4) with Fisher exact test.

**Figure 2:**
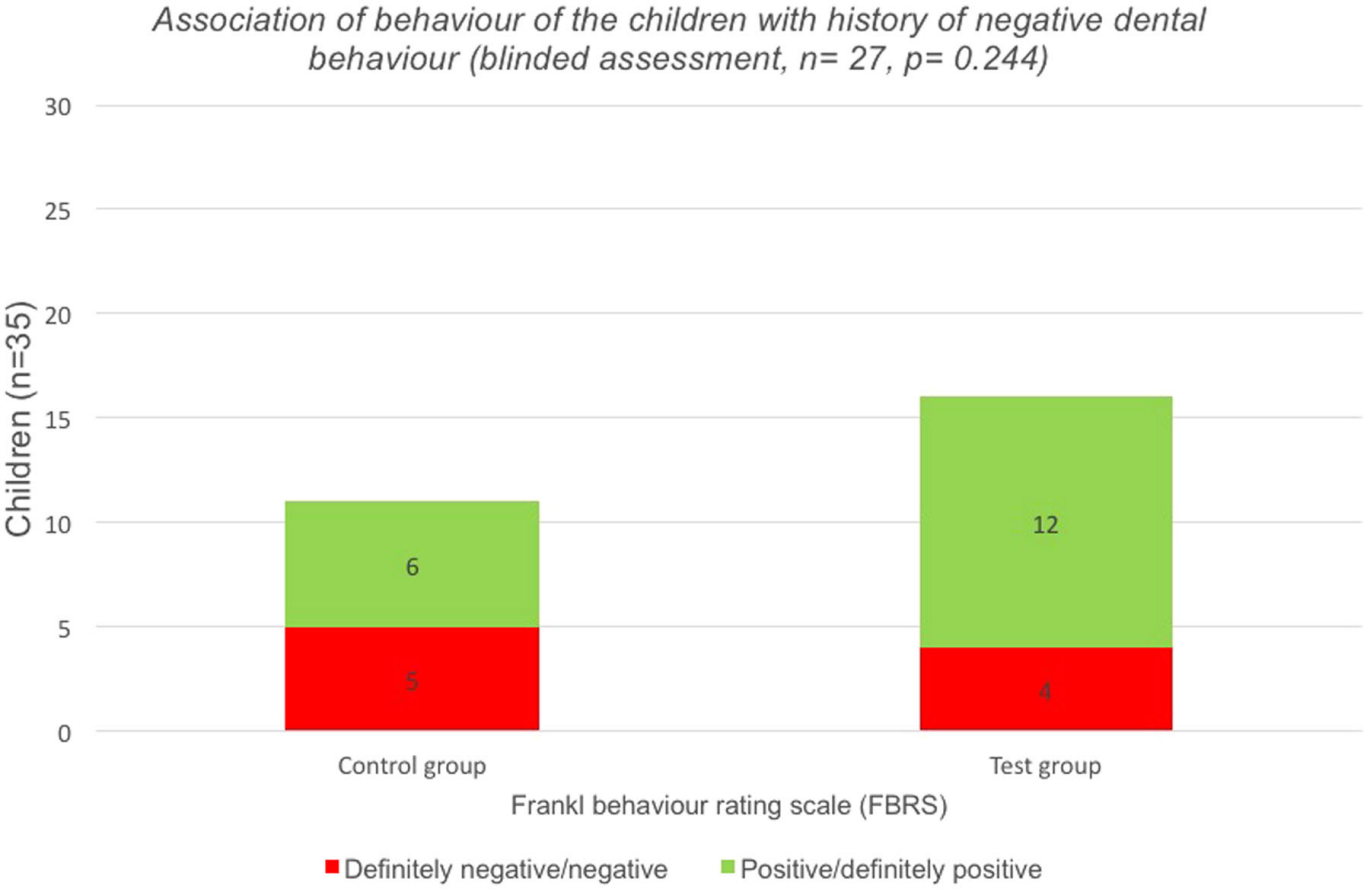
Association of behavior of the children according to FBRS with a history of negative dental behavior in both study groups (blinded assessment, *n* = 27).

Interestingly, a clinically relevant correlation of FBRS was found between children with versus without history negative dental behavior documented in the digital dental records (Table 5), through which among children with negative dental behavior history, the test group was more likely to report positive behavior than the control group (66.6% vs. 33.4%, *p* = 0.244) as shown in Figure 2. On the other hand, there were no statistically significant associations (*p* > 0.05) between test and control group regarding the history of invasive dental procedures, age, and dmft‐index (Table 5).

**Table 5:** Association of behavior of the children (blinded FBRS rating) with baseline characteristics of the study groups (*n* = 70).

Category	Factor	Frankl Behavior Rating Scale (FBRS)	Study groups		Total	Stat.
			Control	Test	*n* (%)	*p*‐value
**History of negativedental behavior** (dental records)	Yes	1 and 2	5 (45.5%)	4 (25.0%)	9 (12.9%)	
		3 and 4	6 (54.5%)	12 (75.0%)	18 (25.7%)	
		***Total***	***11 (16.0%)***	***16 (23.0%)***	***27 (38.6%)***	***p*** = ***0.244*** a
	No	1 and 2	2 (8.3%)	1 (5.3%)	3 (6.9%)	
		3 and 4	22 (91.7%)	18 (94.7%)	40 (93.1%)	
		***Total***	***24 (27.1%)***	***19 (34.3%)***	***43 (61.4%)***	***p*** = ***0.629*** a
**History of invasivedental procedures** (dental records)	Yes	1 and 2	5 (26.3%)	5 (22.7%)	10 (14.3%)	
		3 and 4	14 (73.6%)	17 (77.3%)	31 (34.2%)	
		***Total***	***19 (14.3%)***	***22 (34.2%)***	***41 (58.5%)***	***p*** = ***0.536*** a
	No	1 & 2	2 (12.5%)	0 (0.0%)	2 (2.8%)	
		3 & 4	14 (87.5%)	13 (100.0%)	27 (38.6%)	
		***Total***	***16 (21.4%)***	***13 (24.3%)***	***29 (41.4%)***	***p*** = ***0.296*** a
**Age (years)**		1 & 2	6 (± 1.16)	6 (± 1.41)	6 (± 1.66)	
Mean ( ± SD)		3 & 4	7.1 (± 1.57)	7.6 (± 1.79)	7.34 (± 1.69)	
		***Total***	***6.9 (*** ± ***1.6)***	***7.3 (*** ± ***1.7)***	***7.1 (*** ± ***1.6)***	***p*** = ***0.057*** b
**Mean dmft**		1 & 2	5.8 (± 4.48)	4 (± 2.74)	5.08 (± 3.53)	
(dmft ± SD)		3 & 4	3.6 (± 4.01)	4.1 (± 2.79)	3.88 (± 3.49)	
		***Total***	***4.03 (*** ± ***3.50)***	***4.09 (*** ± ***3.50)***	***4.06 (*** ± ***3.49)***	***p*** = ***0.501*** b

^a^ Exact Fisher test.

^b^ ANOVA single‐factor test.

**Figure 3:**
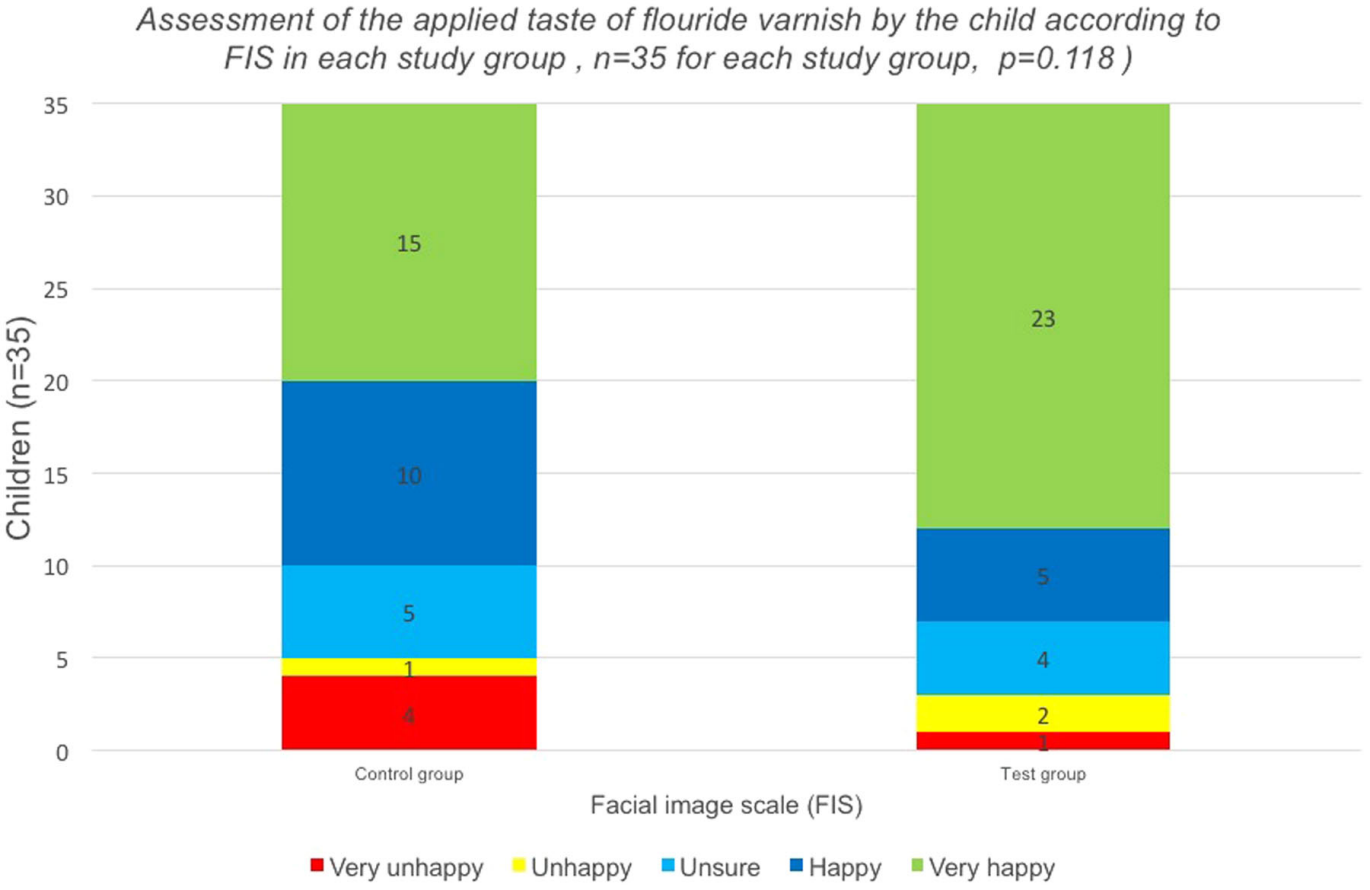
Assessment of the applied taste of fluoride varnish by the child according to FIS in each study group (*n* = 35 for each study group).

FIS assessment revealed statistically insignificant difference between the two groups (*p* > 0.05) as depicted in Table 6. However, the control group had a higher proportion of children (*n* = 4), who felt very unhappy with the allocated taste of fluoride than the test group (*n* = 1). The test group, who had the opportunity to choose the taste was mainly and more often very happy with the taste (Test: 65.7% vs. Control: 42.9%; Table 6). Moreover, according to subgroup analysis, the proportion of children with a history of negative dental behavior within the test group, who reported a positive taste acceptance was higher than in the control group (75% vs. 54.6%, *p* = 0.198), as shown in Table 7 (Figure 3).

**Table 6:** Assessment of the applied taste of fluoride varnish by the child according to FIS (*n* = 70).

Facial imagescale (FIS)	Study group		Total	*t*‐test
	Control *n* = 35 (50%)	Test *n* = 35 (50%)	*n* = 70 (100%)	*p*‐value
**Very unhappy (1)**	4 (11.4%)	1 (2.9%)	5 (7.1%)	*p* = 0.118
**Unhappy (2)**	1 (2.9%)	2 (5.7%)	3 (4.3%)	
**Unsure (3)**	5 (14.3%)	4 (11.4%)	9 (12.9%)	
**Happy (4)**	10 (28.6%)	5 (14.3%)	15 (21.4%)	
**Very happy (5)**	15 (42.9%)	23 (65.7%)	38 (54.3%)	
**Mean (± SD)**	**3.88 (** ± **1.22)**	**4.34 (** ± **1.22)**	**4.11 (** ± **1.22)**	

**Table 7:** Association of taste acceptance (Facial imaging scale) by the child with baseline characteristics of the study groups (*n* = 70).

Category	Factor	Facial imagingscale (FIS)	Study group		Total	Stat.
			Control	Test	*n* (%)	*p*‐value
**History of negativedental behavior** (dental records)	Yes	(1–3)	6 (50.0%)	4 (26.6%)	10 (14.3%)	
		(4–5)	6 (50.0%)	11 (74.4%)	17 (24.3%)	
		***Total***	***12*** **(** ***17.2%*** **)**	***15*** **(** ***21.4%*** **)**	***27*** **(** ***38.6%*** **)**	***p*** = ***0.198*** a
	No	(1–3)	5 (20.8%)	2 (10.5%)	7 (10.0%)	
		(4–5)	19 (79.2%)	17 (89.5%)	36 (51.4%)	
		***Total***	***24*** **(** ***34.3%*** **)**	***19*** **(** ***27.1%*** **)**	***43*** **(** ***61.4%*** **)**	***p*** = ***0.581*** a
**History of invasivedental procedures** (dental records)	Yes	(1–3)	6 (31.6%)	5 (22.7%)	11 (5.7%)	
		(4–5)	13 (68.4%)	17 (77.3%)	30 (42.9%)	
		***Total***	***19*** **(** ***21.4%*** **)**	***22*** **(** ***25.7%*** **)**	***41*** **(** ***55.6%*** **)**	***p*** = ***0.381*** a
	No	(1–3)	4 (25.0%)	2 (15.4%)	6 (8.5%)	
		(4–5)	12 (75.0%)	11 (84.6%)	23 (32.9%)	
		***Total***	***16*** **(** ***21.4%*** **)**	***13*** **(** ***24.3%*** **)**	***29*** **(** ***41.4%*** **)**	***p*** = ***0.435*** a
**Age (years)**		(1–3)	7 ( ± 2)	6 ( ± 1.41)	6.88 ( ± 1.64)	
Mean (± SD)		(4–5)	6.8 ( ± 1.49)	7.5 ( ± 1.79)	7.18 ( ± 1.70)	
		***Total***	***6.9*** **(** ± ***1.6*** **)**	***7.3*** **(** ± ***1.7*** **)**	***7.1*** **(** ± ***1.6*** **)**	***p*** = ***0.241*** b
**Mean dmft**		(1–3)	6.8 ( ± 1.68)	4 ( ± 2.74)	4.52 ( ± 3.50)	
(dmft ± SD)		(4–5)	3.9 ( ± 2.66)	4.1 ( ± 2.79)	3.9 ( ± 3.49)	
		***Total***	***4.03*** **(** ± ***3.50*** **)**	***4.09*** **(** ± ***3.50*** **)**	***4.06*** **(** ± ***3.49)***	***p*** = ***0.981*** b

^a^ Exact Fisher test.

^b^ ANOVA single‐factor test.

## 4 Discussion

This study was conducted as an attempt of strengthening the scientific evidence around the use of a sense of control for motivation of children toward the dental appointment through a single‐blinded randomized clinical trial. A tendency of a more positive behavior during the dental check‐up appointment resulted in a better acceptance of taste of fluoride varnish, which was observed in test group. This positive effect was though more pronounced in children with a history of negative dental behavior but also did not reach a statistically significant difference.

Furthermore, in this study history of negative dental behavior (*n* = 27, 39%) was found to be prevalent among pediatric dental patients. This is relevant since, children with negative dental behavior have a significant higher dental fear compared to those with positive dental behavior (Alshoraim et al. 2018). Nonetheless up to now, there have been few reports/RCTs on this basic behavior management option (locus of control) use in dental setting especially in pediatric dentistry (Enkling, Marwinski, and Jöhren 2006). No reports in the literature are available on the effect of choice of highly concentrated fluoride varnishes with different tastes on acceptance in children and their dental behavior during the treatment in schoolchildren. To fill this gap, the present study evaluated the motivational effect of choosing the taste of a recommended fluoride varnish (22,600 ppm Profluorid, VOCO GmbH, Germany) in a standardized way (RCT).

To measure the dental behavior during the treatment, a single calibrated blinded assessor rated the cooperation, thus reducing investigator bias and preventing subject knowledge of the treatment assignment from confounding study outcomes (Alwin, Murray, and Britton 1991). Dental behavior was assessed by the simple FBRS due to its high 93.4% sensitivity (93.4%) and acceptable validity (77.8%) (Asokan et al. 2014). However, a major shortcoming of FBRS is lack of clear classification with definite items for observation and insufficient description to the specific type of children's negative behavior (Silva et al. 2020). Therefore, additionally the acceptance of the taste was recorded according to self‐report data (i.e., FIS) as the accuracy of children self‐rated scales (FIS) is found to higher than parental assessment (Paglia et al. 2017). The grayscale version was used in this study to avoid any color bias that could accompany colored Facial Scale Index (Buchanan and Niven 2002).

The age of the participants in this study was set in the range of 5–10 years, which is known as the preoperational phase. This phase prepares children for proper social and interpersonal communication due development of attention and cognitive abilities. Thus, they are able to express themselves and more prone to be affected by motivational approaches (Radhakrishna et al. 2019).

However, both study groups behaved positively during the dental check‐up appointment, in which a fluoride varnish (VOCO Profluorid, Germany) with various tastes was used (Table 3). Reason behind this might be the underlying study sample, who consisted of healthy, symptom‐free children, presenting for a routine dental check‐up. It is known that a significant correlation between negative dental behavior in children with experience and symptoms of toothache (Dahlander et al. 2019) which could also be seen in this data.

This study was performed in a specialized Paediatric dental department of university medicine Greifswald (Germany). To reduce the risk of bias in the performance of the treatment, the dental check‐ups were done by multiple persons either by a specialized pediatric dentist or postgraduate student, who were taught on the same clinical concept of behavioral management techniques. Previous studies showed that professional training plays an important role in choosing the suitable behavioral management techniques in children (Folayan and Idehen 2005).

In general, a pleasant taste is crucial for the acceptance of dental products used for caries prevention (Autio and Courts 2001). This study revealed a tendency for children to prefer fluoride varnishes with pleasant tastes, though the mint taste was the least preferred. This is also supported by other studies suggesting an inherent preference of a sweet taste rather than sour taste (Dovey et al. 2008; Keskitalo et al. 2007).

The main result of the present study observed a clinically relevant outcome, in which the proportion of children with a history of negative dental behavior, who showed a positive behavior in the test group was 2.5 times more than the control group (Table 5). Moreover, patients who had a history of invasive dental procedures showed improved dental behavior during dental visit, in which an application of fluoride varnish was performed. These results of the present study are in compliance with similar studies, which involved the use of pseudo‐alternatives (locus of control) for the color of dental restoration. A better acceptance of the dental treatment was reported in children, who chose the color of dental restoration themselves. Thus, this approach was considered an effective motivational tool, where children outlook the dental appointment as an interactive experience (Fishman, Guelmann, and Bimstein 2007; Rank et al. 2019). Offering the sense of control, as in this case choosing the taste of fluoride varnish can have positive effects. Children who seem tense after operative dental procedures may suddenly become happy when receiving a self‐chosen material and positive verbal reinforcement, thus representing a pleasant memory of the dental visit (Rank et al. 2019). A recent study reported a significant preference for flavored dental materials in which, flavors were closely linked to the child's emotions, suggesting that they can serve as an effective tool in managing the child's behavior (Ashwin and Jessy 2020). Thus, positive changes in children's behavior are not only beneficial to reduce the degree of dental anxiety but also contribute to oral health improvement, hence facilitate the implementation of caries prevention in high‐risk children (Cianetti et al. 2017).

The main result of the study supports the clinical impact of high autonomous and low controlling treatment styles carried out at dental clinics, as perceived by patients, which were reported to reduce dental anxiety, improve dental attendance and lower avoidance of clinic appointments. Thus providing options and a rationale for change, elicit and reflect on patient perspectives, support patient's initiatives, minimize pressure and a controlling language, which hence improves child's acceptance toward the treatment (Halvari et al. 2020).

In the present clinical trial, the application of fluoride varnish with various tastes was assessed positively in children of both study groups (*n* = 53, 75.7%). The study results support applicability of providing pseudoalternatives (locus of control) in pediatric dental practice due to its simple, safe, low cost and time‐saving implementation especially in children with history of negative dental behavior or it can be used in conjunction with other behavioral management technique.

One limitation of the study is the sample size, though including already 70 participants. Most likely the assumed difference in behavior between both groups was a little bit too high. A larger sample size or a modification of inclusion criteria by only including children with a history of negative dental behavior and potentially cooperative children based on Wright's classification of cooperativeness of children in this specialized pediatric dental setting might have elucidated the differences of the effect of the choice on behavior (Kupietzky 2022). Nonetheless, the direction of the effects is shown and this study is one of few providing scientific evidence and not only clinical experience to support the use of the behavior management technique “Sense of Control” in daily office.

To conclude, choosing the flavor of the fluoride varnish may increase the chance for positive behavior during the dental visit especially in children with history of negative dental behavior. Therefore, sense of control as an easy and quick behavior guidance technique may be further advocated in daily clinical practice.

## Clinical Relevance

Permitting the child to choose through sense of control, for example, the taste of the fluoride varnish is a simple technique at basically no cost, which may provide a positive influence on children with history of negative dental behavior. Thus, choosing the taste of fluoride varnish not only contributes to positive dental behavior during the dental check‐up appointment but also showed a clear tendency to enhance the children's attitude and acceptance toward the application which is important for dental caries preventive measures in the long‐run.

## Author Contributions

R. Karim, C. H. Splieth, and J. Schmoeckel conceived the ideas and designed the study. R. Karim and J. Schmoeckel collected and analyzed the data; and R. Karim and J. Schmoeckel led the writing and drafted the manuscript with guidance and revisions from C. H. Splieth. All authors critically appraised, revised, read and approved the final version of the manuscript as submitted.

## Ethics Statement

This randomized, prospective study was approved by the Local Ethics Committee (BB 180/21) and was conducted in strict adherence to the 2010 CONSORT statement.

## Consent

All parents/caregivers were asked to sign an informed consent after thorough explanation of the procedures and possible outcomes of treatment. Children were excluded from the study when their parents declined to sign the form.

## Conflicts of Interest

The first author received for his postgraduate master's project a low budget financial support from VOCO GmbH, Germany. All authors declare no potential conflicts of interest with respect to authorship and/or publication of this article.

## Supporting information

Supporting information.

## Data Availability

The data set used in this study contain sensitive details of pediatric patients, which can't be uploaded to an open repository for ethical reasons.
